# A Rare Presentation of Small Bowel Obstruction Due to Obstructed Indirect Inguinal Hernia

**DOI:** 10.7759/cureus.20656

**Published:** 2021-12-24

**Authors:** Hany A Zaki, Eman E Shaban, Adel Zahran, Khalid Bashir, Amr Elmoheen

**Affiliations:** 1 Emergency Medicine, Hamad Medical Corporation, Doha, QAT; 2 Cardiology, Al Jufairi Diagnosis and Treatment, Doha, QAT; 3 Medicine, Qatar University, Doha, QAT

**Keywords:** operative laparoscopy, small-bowel obstruction, obstipation, abdominal wall, indirect inguinal hernia

## Abstract

Inguinal hernia is the most common hernia that affects the anterior abdominal wall. It is important to note that increased intra-abdominal pressure is a major risk factor of inguinal hernia formation. An indirect inguinal hernia in the right groin region causing small bowel obstruction is rare in elderly females. The current report illustrates the case of a 53-year-old female presenting with a history of abdominal pain (colicky) with vomiting and nausea, which required diagnostic laparoscopy plus open mesh repair of right inguinal hernia. Operative findings reveal a hernia in the right groin extended to the right labia containing fat and a segment of distal ileum that shows decreased wall enhancement with the surrounding fluid, leading to small bowel dilatation up to proximal jejunum with a maximum diameter of about five cm. This case highlights the importance of obstipation imperative to the diagnosis of small bowel obstruction due to obstructed indirect inguinal hernia.

## Introduction

Inguinal hernia is the most common hernia that affects the anterior wall of the abdomen [1-3]. Inguinal hernia may be caused by either congenital or acquired factors. Acquired factors are multifactorial and may be due to inherent weakness of the genetic fascia after altered hydroxylation of hydroxyproline in type 1 collagen, or secondary to self-induced factors such as steroid usage, smoking, and increased pressure of the abdomen [4]. Increased intra-abdominal pressure is also a risk factor for the formation of hernia depending on the amount of stress that the inguinal canal's shutter mechanism is subjected to, as well as the possibility of the increased intra-abdominal pressure overloading the protective shutter mechanism [4]. The failure of this protective mechanism results in widening of the internal ring whether or not there is a defect in the transversalis fascia. Having an in-depth knowledge of the genesis of inguinal hernia is vital to knowing the most effective treatment modality and preventing a recurrence.

Less than five percent of women ever experience inguinal hernia [5]. Although it may be infrequent, hernias, when present, must undergo urgent evaluation due to possible strangulation or incarceration of organs, including, in very rare cases, the fallopian tube and the ovary. In most cases, an inguinal hernia can be diagnosed based on physical examination and history alone. These hernias may present as asymptomatic findings like a non-painful bulge in the region of the groin, or in an acute or subacute manner, with pain in the area of the abdomen-pelvis [6]. When an elderly female presents with severe pain in the pelvic pain, hernias of the abdominal wall must be considered in the differential. There is a high chance of hernias being frequently overlooked, especially if it is not assessed in a physical examination.

These hernias may contain several visceral organs, with omentum or intestines being found commonly, resulting in strangulation or incarceration, which would be considered a surgical emergency. There may be an entrapment of the fallopian tube or ovary, though this happens in very rare cases. The patient presentation may be in a nondescript manner describing a dull discomfort or heaviness in the groin area that is most pronounced during an increase of the intra-abdominal pressure. Typically, diagnosis of hernias may be made by careful palpation on physical examination if considered and verified by ultrasound imaging. It is important to note, though, that when results from an ultrasound scan remain ambiguous and there is fear of organ entrapment, it is important to carry out a CT scan for the purpose of definitive diagnosis and to aid proper counseling, consultation, and surgical management.

We present the case of a female patient diagnosed with a complicated hernia in the right groin region, causing small bowel obstruction.

## Case presentation

A fifty-three-year-old female patient presented to our facility with a history of abdominal pain (colicky) with nausea and vomiting after a heavy meal. She denied any abnormal urinary or bowel habitus. The symptoms persisted for four days despite symptomatic treatment in the form of 20mg scopolamine intramuscular (IM) injection once, 10mg oral domperidone tab (seven days), 40mg simethicone oral capsules three times a day (TID) for seven days. She visited the health center due to persistent symptoms plus abdominal distention. The patient had a past medical history of irritable bowel syndrome. There was no previous surgical intervention. There was also no recent hospital admission or significant allergy.

**Figure 1:**
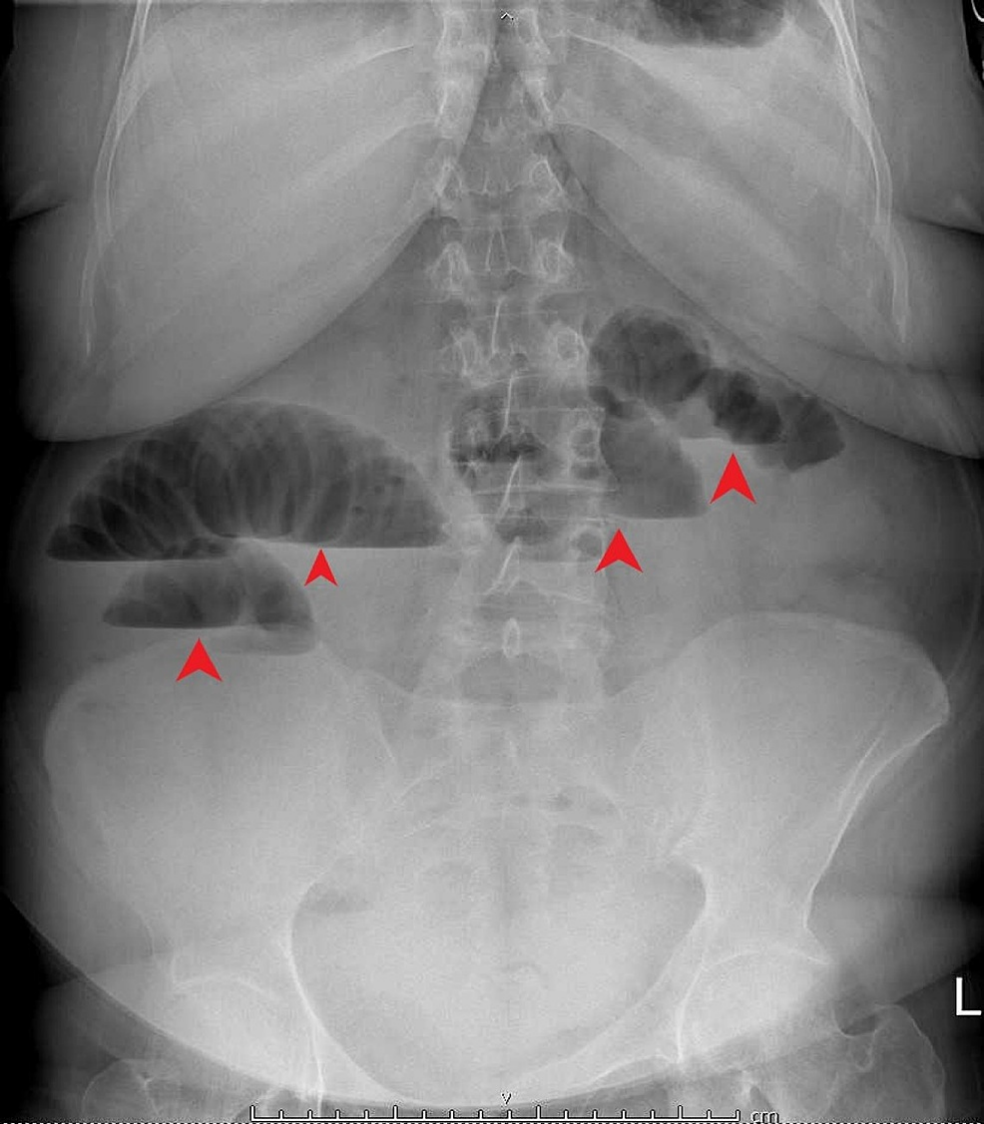
Abdominal X-ray (red arrowheads) demonstrate dilated bowel loops (likely small bowel) with multiple fluid level noted suggesting small bowel obstruction

An abdominal X-ray (XR) was ordered at the health center (Figure 1), after which she was referred to the emergency department for further management and assessment.

Her temperature was taken orally and showed 37 degrees Celcius, peripheral heart rate was a high 134 bpm, respiratory rate was 18 br/min., systolic blood pressure (SBP) was 122 mmHg, diastolic blood pressure (DBP) was 83 mmHg., and oxygen saturation levels (SpO2) were 96%.

Physical examination

General Examination

**Figure 2:**
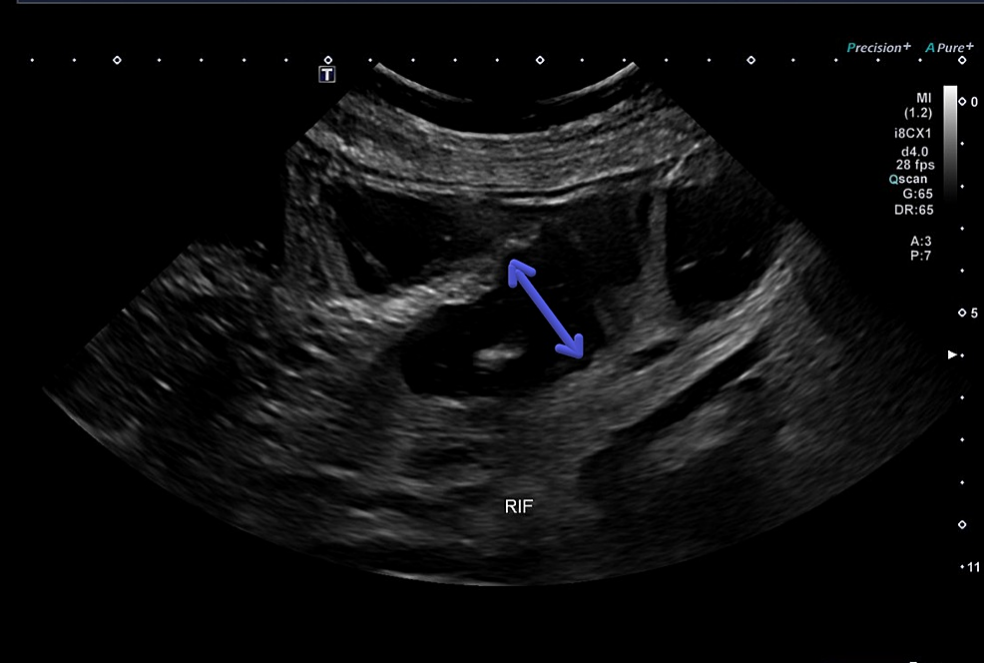
Ultrasound view image demonstrate prominent bowel loops are noted (double-headed arrow), with to and fro movement detected during the ultrasound examination

The patient appears ill and dehydrated. An examination of the patient's cardiovascular system showed a regular pulse with normal character, no elevated jugular venous pressure (JVP), normal first and second heart sounds, no murmurs, and no pedal edema. Gastrointestinal (GI)/ genitourinary (GU) system & abdominal examination showed bluish discoloration on the umbilical area, a profusely distended abdomen, no organomegaly, mass on the right inguinal fossa. Diminished bowel sounds, hernial orifices were not intact at the right inguinal area with mild diffuse abdominal tenderness, tenderness on the right lower quadrant (RLQ) with mild guarding and rigidity. An ultrasound image of the abdomen is shown in Figure 2.

**Figure 3:**
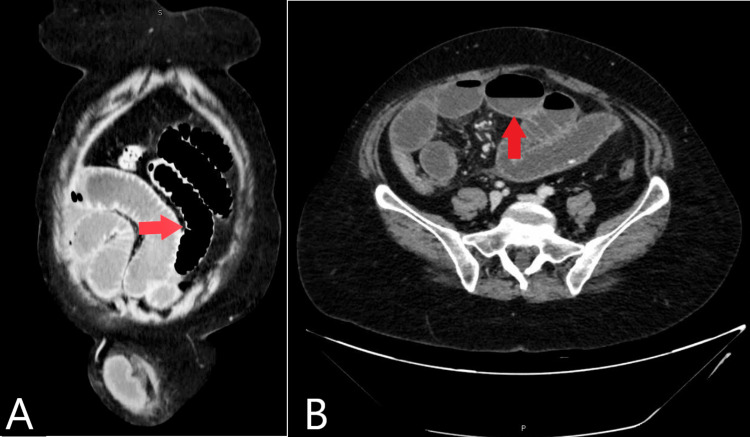
CT of the abdomen and pelvis (A) Coronal view with red arrow to demonstrate small bowel obstruction up to proximal jejunum. (B) Sagittal view with red arrow to demonstrate multiple air-fluid levels suggest small bowel obstruction.

**Figure 4:**
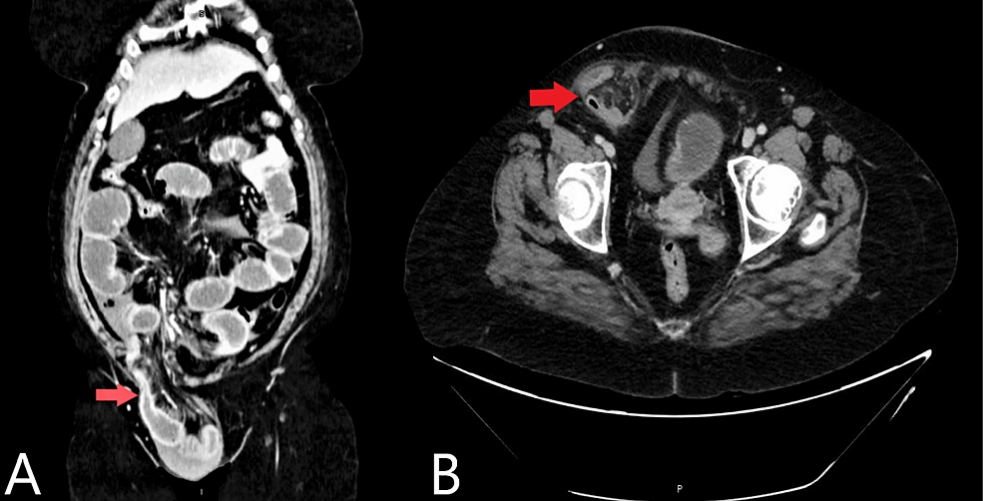
CT of the abdomen and pelvis (A) Coronal view with red arrow to right inguinal hernia. (B) Sagittal view with red arrow to demonstrate right-sided inguinal hernia extending to the right labia suggesting of complicated right inguinal hernia.

Later highly suspicious of intestinal obstruction was made according to the clinical and ultrasound imaging, so computed tomography with the contrast of the abdomen and pelvis was done with the following results (Figures 3, 4).

Management

Surgical repair of hernia was once considered to be controversial due to accompanying complications whether infection, incarceration, or strangulation. But recently it was proven that asymptomatic hernias occurring for the first time do not need repair, but to follow up for aforementioned complications. In case there was deterioration or recurrence of hernias, it should be referred for repair. Surgical options vary in respect of individual circumstances, including the location of the hernia. There are two main types of surgical intervention for hernia: open surgery and laparoscopic repair (which has the advantage of being less likely to cause infection). Here, the patient was admitted under general surgery as a case of obstructed right inguinal hernia with small bowel obstruction. The surgery uncovered obstructed right indirect inguinal hernia, hyperemic congested viable small bowel loops in the hernial sac, dilated small bowel loops, and hemorrhagic fluid in the pelvis.

Design

Operation is done with diagnostic laparoscopy plus open mesh repair of right inguinal hernia, the estimated blood loss was 20 ml and urine output was 170 ml.

Method

With the following technique under general anesthesia, the patient was draping in the supine position. Pneumoperitoneum was identified and treated by open technique via 10 mm supraumbilical port, two 5 mm ports insertion under vision in left umbilical & left inferior fossa. Hernia contents (small bowel loops) were reduced, the viability of the herniated loops was confirmed (color improved after 100% oxygenation, peristaltic movements & mesenteric pulsation noted). Bloody fluid collection was suctioned, drain size 19 was placed in the pelvis. Open mesh repair of the right inguinal hernia was performed after excision of the hernial sac. Hemostasis was controlled, and the drain was placed over the mesh. Sharps & swabs count was found to be correct as per the scrub nurse. Ports were removed under vision. The closure was done through the umbilical port with J needle vicryl 0, left umbilical port with 3/0 monocryl, the inguinal wound was closed in layers, and pressure dressing applied.

Outcome

The patient was discharged after five days of hospital admission on oral metronidazole, NSAIDs, and follow-up to the general surgery clinic after one week.

## Discussion

There are two types of inguinal hernias: direct hernias and indirect hernias. Direct hernias, a protrusion occurs through Hesselbach's triangle, which is medial to the inferior epigastric vessels. Indirect hernias, on the other hand, feature a protrusion through the inguinal ring, typically lateral to inferior epigastric vessels [6]. The prevalence of indirect hernias is higher than that of direct hernias in adult women. Indirect hernias in adult women typically occur between the age of 40-60 [5]. There are intestinal contents in a large percentage of these hernias and rarely viscera as in female adnexa (fallopian tubes or ovaries) in over three percent of cases [7]. If the female adnexa is entrapped, it is more common in infants owing to anatomical causes such as a sort inguinal canal, a canal obliquely directed through the wall of the abdomen, and a Nuck diverticulum (a pocket of peritoneum that is linked to the round ligament corresponding to the vaginal processus in infant males) [7-10].

On the other hand, entrapment of the adnexa within an indirect hernia would be uncommon and unexpected. When present in adult females, most reported hernias are usually seen in postmenopausal or perimenopausal women [7,11]. But one must take into consideration the differential in premenopausal women. In contrast to the current case, previous case reports of reproductively active women with entrapped adnexa have been linked with fallopian tube defects, such as ovarian hemorrhagic cysts or paratubal cysts, which may trigger a weighted organ descent and thus predispose for entrapment [11,12]. Other factors that may constitute a risk include lengthening of the suspensory ligament of the ovary or uterus in high parity patients leading to the displacement of structures in the adnexa as well as high intra-abdominal pressure from excessive Valsalva maneuvers in people with frequent heavy lifting or chronic cough [9]. The patient, in our case, did not experience any enlargement of the adnexa or pathology identified from the aforementioned risk factors.

Timely management is important to ensure effective surgical intervention to prevent or minimize damage to the ovary and subsequent infertility. Of course, there will be a need for collaboration by general surgeons and gynecologists. Both parties must also recognize this condition to ensure that fertility is preserved. In previous reports featuring the involvement of the female adnexa, more than 50% had the need for oophorectomy after strangulation [7]. After extending to cases that involved children or female infants, 27% of those presenting with irreducible ovaries had infarcted ovaries during surgery [10]. Adnexa incarceration on its own may decrease the supply of blood. Blood flow would be further compromised with torsion. Thus, female adnexa is especially vulnerable to damage upon entrapment in inguinal hernias, and the inability to recognize this may result in infarction of the fallopian tube/ovary.

## Conclusions

Indirect inguinal hernia, though rare, should be suspected in individuals with a large, incarcerated hernia, where there is evidence of peritonitis or strangulation, or where there are viable loops of the intestine in the hernial sac. Bowel loops that lie proximal to the obstructing hernial ring should be examined as a means of preventing non-viable bowel from returning to the abdomen during the repair. Also, diagnosis of adnexa and other entrapped viscera should be taken into consideration during differential diagnosis of hernias in females of adult age. This will guarantee timely and defective medical and surgical management to ensure that patient's fertility is preserved. Timely surgical intervention is important to relieve and prevent torsion and to restore adequate perfusion to the adnexa. Diagnosis may be enhanced by multiple imaging studies such as cross-sectional imaging via CT or ultrasound.
